# The Pressure induced by salt crystallization in confinement

**DOI:** 10.1038/srep30856

**Published:** 2016-08-05

**Authors:** J. Desarnaud, D. Bonn, N. Shahidzadeh

**Affiliations:** 1 Van der Waals-Zeeman Institute, Institute of Physics, University of Amsterdam, Science Park 904, 1098 XH Amsterdam, The Netherlands

## Abstract

Salt crystallization is a major cause of weathering of rocks, artworks and monuments. Damage can only occur if crystals continue to grow in confinement, i.e. within the pore space of these materials, thus generating mechanical stress. We report the direct measurement, at the microscale, of the force exerted by growing alkali halide salt crystals while visualizing their spontaneous nucleation and growth. The experiments reveal the crucial role of the wetting films between the growing crystal and the confining walls for the development of the pressure. Our results suggest that the measured force originates from repulsion between the similarly charged confining wall and the salt crystal separated by a ~1.5 nm liquid film. Indeed, if the walls are made hydrophobic, no film is observed and no repulsive forces are detected. We also show that the magnitude of the induced pressure is system specific explaining why different salts lead to different amounts of damage to porous materials.

Salt weathering affects porous materials such as rock outcrops and minerals within the soil profile but also engineering structures monuments, and artworks (Fig. 1). There is compelling evidence that its influence will increase due to global climate change^1^,^2^. Refs 1 and 2 underline the great importance of atmospheric moisture change as a threat to heritage, despite the fact that often only temperature is identified as the key aspect of climate change. The risk factors related to such a change include sea level rise, intense rainfall, flooding and changes in humidity cycles which can considerably amplify phase changes in freeze-thaw cycles and salt crystallization. In the case of sodium chloride contaminated stones, it has been also shown how humidity fluctuations (i.e. deliquescence followed by drying) induce the recrystallization of large NaCl crystals in the pores at the subsurface of the porous material leading to damage after several cycles^3^.

Moreover, changes in humidity affect also the growth of microorganisms in stone and wood^2^. Drier summers overall, for instance, may increase salt weathering of stone, and desiccate the soils that protect archaeological remains and support the foundations of buildings. In this way, many ancient structures and historical artworks, such as the Valley of the Kings (Egypt), the Petra site (in southern Jordan), and a large number of frescos and sculptures have already been partially or even completely destroyed by salt attack^4^,^5^,^6^,^7^,^8^,^9^,^10^,^11^,^12^.

Arguments explaining that growing crystals can generate stresses have been given for more than 150 years ago, since Lavalle and subsequently Becker reported that a crystal can grow under external pressure^13^,^14^. They assumed that the crystal could grow and overcome the external forces provided the surface is in contact with supersaturated solution^15^,^16^,^17^,^18^. In its simplest form, the crystallization pressure P_crystallization_ was approximated by the chemical potential difference between salt molecules in the crystal and in the supersaturated salt solution:
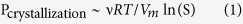
with ν the number of different ions, R the gas constant, T the temperature, V_m_ the molar volume of the solid phase, and S the supersaturation, which is the ratio of the solute concentrations in the solution *c* and in the saturated solution *c*_*s*_^16^,^17^,^18^,^19^,^20^.

More formally, this requires an activity product K[P] greater than the solubility product at atmospheric pressure K[0], as described by Freundlich equation for a crystal under pressure 
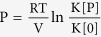
. Although several such theoretical corrections have been made on this equation by taking into account the water activities and the size of the crystal^17^,^19^,^20^,^21^,^22^, it does not fully explain the different experimental results reported in literature, notably the very different weathering effect of different salts.

The direct measurement of the force exerted by a growing crystal in confinement is challenging, as illustrated by the small number of experimental results reported^16^,^23^,^24^,^25^,^26^. Previous studies consider the growth of a pre-existing crystal confined between two plates under a load and immersed in a salt solution. On the one hand, some evidence that a crystal under stress can grow has been reported^25^, in addition to a displacement of the wall due to crystal growth^15^,^16^ or a repulsive force between a crystal and a wall using AFM^26^. On the other hand, other groups have reported the dissolution of the loaded face and the growth of unloaded ones^23^,^27^: a crystal in a supersaturated solution can simply grow in a direction in which it is not confined and not exert a pressure. Therefore, the debate about the mechanism involved in the development of such a pressure continues.

We report experimental results obtained with an innovative setup which permits to visualize the spontaneous primary nucleation and growth during the evaporation of the salt solution while directly measuring the force exerted by a crystal during its growth in confinement. Here, we do not impose a certain supersaturation, or impose a given load; we rather measure the force that the growing crystal exerts with its spontaneous precipitation. This should be contrasted with earlier attempts to measure the pressure exerted during a secondary nucleation process (growth of pre-existing crystal), for which only very modest supersaturations were considered^23^.

## Experimental Methods and Materials

Two alkali halides salts, sodium chloride (NaCl) and potassium chloride (KCl), are studied firstly because of their abundance and similar crystalline stucture (i.e. cubic rock-salt structure). The second incentive for this choice of materials is that recently in experiments on the evaporation of NaCl solutions it became clear that primary nucleation results in very high supersaturations S = *c*/*c*_*s*_ ~ 1.6 ± 0.2 at the onset of crystallization^28^,^29^. As the exerted pressure scales with ln(S), these recent findings suggest a scenario in which the force exerted by a growing crystal should be more easily detected than in previous experiments.

Our experimental setup is as follows (Fig. 2). A very small drop (V_0_ ~ 0.2 μl) of the salt solution (NaCl or KCl) close to saturation (S = c/c_s_ = 0.9, with 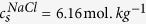, 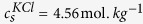) is deposited on a cleaned glass slide on an inverted microscope. The second glass slide that is used to confine the drop from above with a microscale gap is attached to a mechanical testing machine (a rheometer) that allows to (i) control and measure the gap between the plates, (ii) detect the normal force on the plate during the crystal growth, and (iii) deduce the surface area of the salt crystal that is in contact with the plates. The latter is done by applying very small oscillations to the upper plate and measuring the resistance of the material between the two plates to a small shear force. In parallel, by direct visualization using a CCD camera coupled to the inverted optical microscope, we follow the evaporation of the entrapped salt solution and determine the volume change (knowing the diameter of the droplet and the gap between the two plates), the concentration at the onset of the crystal precipitation, and visualize the crystal growth in the solution. Experiments are done at controlled relative humidities of 40% and 4%.

In our climatic chamber (Fig. 2), the relative humidity is fixed by controlling the partial vapor pressure P_w_ entering the set up chamber at the laboratory temperature T_2_ (21 °C)^30^. This is done by flowing air through water at a given temperature T_1_ using a water bath thermostat. Once this air flow arrives in the chamber at the temperature T_2_ (21 °C), the partial vapor pressure P_w_(T_2_) will be then equal to the saturated vapor pressure at T_1_ and consequently the relative humidity of the chamber will be fixed as:
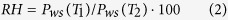
Thus, by changing T_1,_ the relative humidity can be fixed to a desired value in the chamber very precisely (±2%).

## Results and Interpretation

Once the salt solution is entrapped between the two glass plates, an attractive (negative) capillary force (F ~ −3.5 10^−3^ ± 1.0 10^−3^ N) is detected; the measured force is in good agreement with the calculated theoretical value by considering the gap distance, the surface tension of the salt solution and its contact angle with the glass plate^31^ (see Supplementary Information). Upon evaporation of the water, generally a single crystal is observed to form. In the first 5 seconds after its precipitation, the crystal size grows to fill the gap (50 μm) with a film of solution between the crystal faces and the glass plates (Fig. 3).

With the further growth of the crystal, we find that a strong repulsive (positive) force develops, increasing in time. A steep increase of the force is detected when there is no more solution around the sides of the crystal (i.e. no more lateral growth): the remaining salt solution is in the liquid film that is observed between the glass plates and the crystal. Its subsequent evaporation induces the formation of steps on the confined face of the crystal^25^ and the precipitation of small microcrystallites around the main crystal (‘efflorescence’)^32^,^33^ (Figs 3 and 4). For NaCl, the maximum value of the force ΔF ~ 0.03 ± 0.007 N is achieved when the remaining film of salt solution is thinner than the optical resolution of the microscope (Fig. 3).

**Figure 1:**
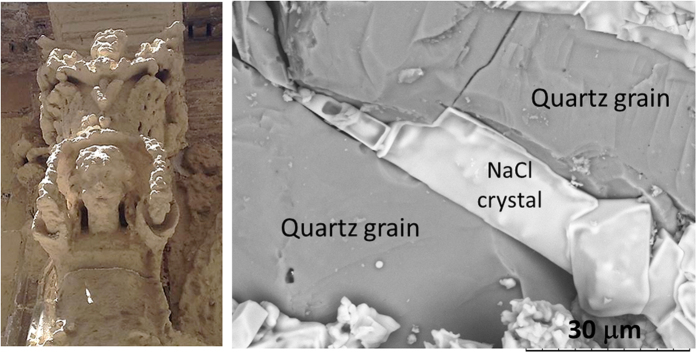
Left: Degradation of a historical stone sculpture (Lecce, Italy). Right: SEM image of NaCl crystals precipitation (white) in the pore space of sandstone after evaporation of salt solution. This image of crystallization in real porous material shows a very similar situation to our microscopy experiment of crystallization between two parallel plates. The two perpendicular microcracks could be due to salt crystallization in the pore space.

**Figure 2:**
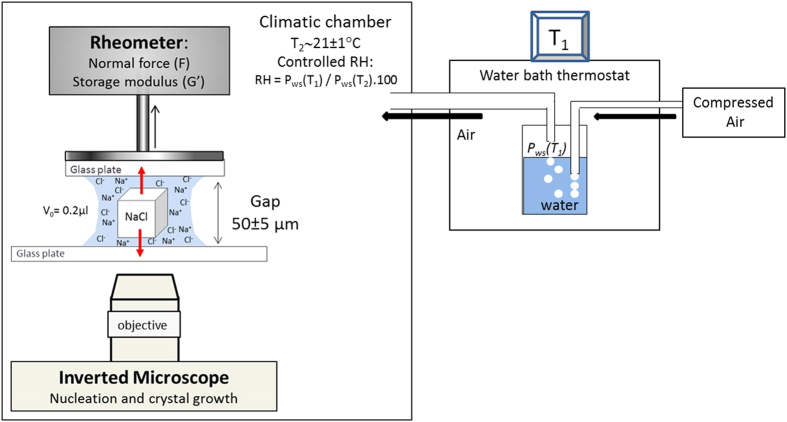
Setup for the visualization of the spontaneous nucleation and growth of a crystal in microscale confinement and the simultaneous detection of the force exerted by the growing crystal. The spontaneous precipitation of the microcrystal is induced by controlled evaporation of the salt solution at temperature T = 21 ± 1 °C at relative humidities RH = 40 ± 3% or RH = 4 ± 2%.

**Figure 3:**
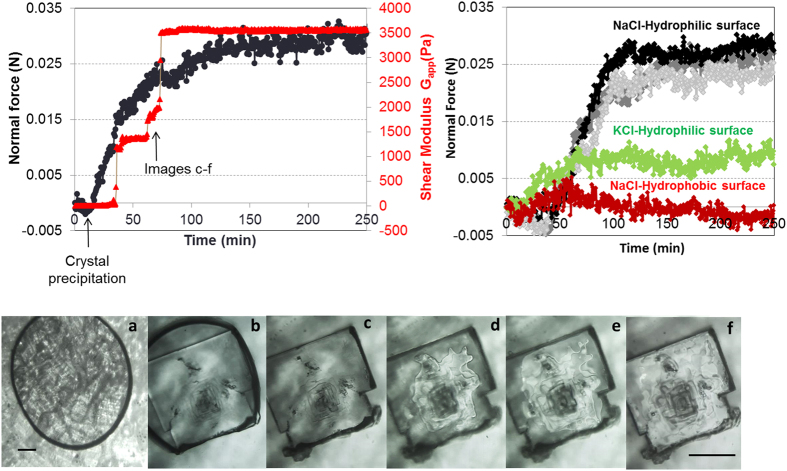
Top Left: Evolution of the normal force and the apparent shear modulus with the NaCl growth from the solution during evaporation. Top Right: 3 experiments of NaCl growth between hydrophilic glass slides showing the reproducibility (**Black and grey curves)**; measured force for a KCl crystal between two hydrophilic glass plates (**green curve**) and on NaCl crystal between two hydrophobic glass plates (**red curve)**. Bottom: evaporation of the NaCl solution entrapped between two hydrophilic glass plates (**a**) before crystal precipitation; (**b**) crystal precipitation and lateral growth. (**c**–**f**) Crystal growth and the drying of the liquid film between the confined crystal face and the glass plates. The variation of the grey level in images e and f is due to the drying of the entrapped liquid film and the evolution of steps in the confined face of the crystal. Development of efflorescence is visible in the left corner of the main crystal (image (**c**–**f**)); Scale bars: 100 μm.

In order to convert the measured force into a pressure, we measure the area of contact between the salt crystal and the glass plates using the rheometer (see Supplementary Information). The latter gives the shear modulus of any solid material that occupies the gap between the two plates, by measuring the shear stress necessary for a certain imposed deformation. The bulk salt solution is liquid and does not resist shear, whereas the salt crystal does^34^. For the evaluation of the area of contact, we use the apparent shear modulus at the end of drying experiments where no more water is left, because the mechanical behaviour of the very thin film between the crystal and the wall is less clear. An independent series of measurements at ~4% relative humidity (i.e. very dry air) show comparable results at the end of drying, showing that the results are not affected by e.g. capillary condensation in the gap between the crystal and the walls. The growth of the crystal is indeed observed to lead to a strong increase of the apparent shear modulus that increases in a similar fashion as the force, although more intermittently. For the experiment shown in Fig. 3, the contact area is A ~ 136 μm^2^; the total area of one of the crystal face is much larger, on the order of ~2–3 10^5^ μm^2^. This then gives a crystallization pressure deduced from the measured force and contact area of about 220 ± 50 MPa for experiment in Fig. 3, a very large pressure that is comparable to the yield stress of NaCl^35^ and is sufficient to damage e.g., the sandstones of Fig. 1^36^,^37^. It is difficult to compare this to earlier AFM measurements of such interactions^26^, since these were done on a different salt in contact with a different wall.

**Figure 4:**
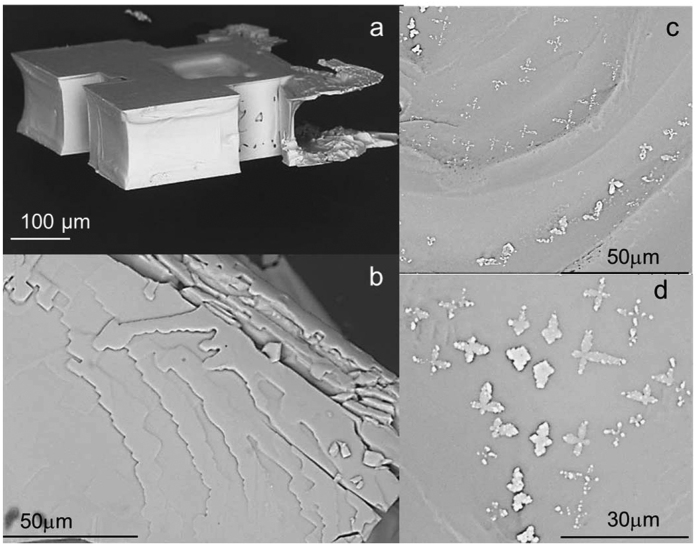
SEM images of NaCl crystals: (**a**) the profile view and (**b**) the confined face of the crystal at the end of the experiment (t = 300 min); (**c**,**d**) The confined face of NaCl crystal half-way through drying (t = 150 min); precipitation of large amount of tiny dentritic crystals caused by the evaporation of the probably highly concentrated film of salt solution.

At the end of each drying experiment, the precipitated microcrystal is gently removed and analysed by SEM; it shows very complex microscale step architecture, in line with the obersvations of Fig. 3(e,f). Surprisingly, the height of the NaCl microcrystals is found to be around 120 ± 20 μm (Fig. 4), i.e. larger than the gap that was imposed between the two plates (~50 μm). This occurs because the confined crystal continues to grow and bends the thin microscope slide that confines it. This explains why a plateau is reached for the measured force at the end of drying; the growth of the micro crystal can be considered as a three point bending experiment. In this case, the estimated magnitude of the force required to make a deformation of about ~50 μm of the glass plate is about F ~ 0.026 N which is of the same order of magnitude as the value of the force achieved at the end of experiment (with 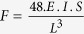 with 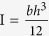; with L = 67 mm, E = 69 10^9^ Pa for glass, the thickness of the glass slide is h = 0.9 mm and a crystal size b ~ 0.8 mm and a deformation S ~ 50 μm). These results obtained at the microscale confirm once again the arguments given at the beginning of the 20^th^ century that a growing crystal in confinement can lift a load if the latter is in contact with a film of solution^14^,^15^.

The importance of thin liquid films for the crystallization pressure was already underlined theoretically^15^,^16^,^17^,^18^,^19^,^20^,^21^,^22^: in order for a crystallization pressure to develop, one needs to maintain a film of liquid by some repulsive forces between the crystal and the wall as this allows for continued crystal growth by the addition of extra ions to the crystal lattice. If a growing crystal fully bridges the gap between two confining walls, no crystallization pressure can be developed since no extra layers of salt can be added to give rise to such a pressure. Subsequently, we have performed the same experiment with glass plates that are made hydrophobic by a silanization treatment. Indeed, in this case, as the formation of a wetting film is prevented, no repulsive force is detected during growth within the experimental uncertainty (Fig. 3). These experimental results make DLVO-type thin-film forces an obvious candidate for the origin of the crystallization pressure^21^,^38^. The Van der Waals forces through a thin film of salt solution between a salt crystal and a glass surface are attractive, and thus could not lead to a pressure pushing the two surfaces apart. A charge repulsion between the two surfaces, provided they have the same charge. If this were the case, the *disjoining pressure* Π(l) within a film of thickness *l*, pushing the two surfaces apart, would be of the (simplified) form Π(l) ~ A exp(−κl), with 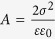, σ the charge density at the surface, ε and ε_0_ the relative permittivity of the bulk and free space, respectively, and 
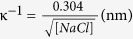
 the Debye screening length with [NaCl] the molar sodium chloride concentration^38^.

**Figure 5:**
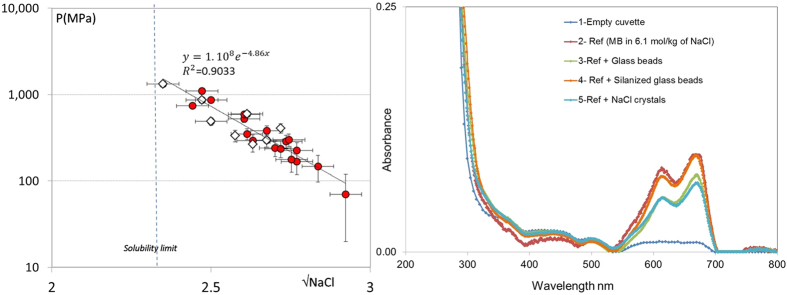
Left: The measured pressure (at the end of the experiment) induced by the spontaneous precipitation of NaCl crystal between two hydrophilic glass plates as a function of the square root of the salt concentration (in Molar) at the onset of crystallization. White square symbols: experiments at RH ~ 4%; red circles: at RH ~ 40%. The solid line is a fit to the expression for the repulsive force between two charged surfaces. Right: UV-Vis Spectrometer experiments for (1) an empty cuvette (2) the reference solution (Ref): The Methylene Blue (MB) in 6.1 mol.kg^−1^ of NaCl solution (3, 4 and 5). The reference solution in contact with glass beads, silanized glass beads and 2 g of NaCl crystals added to the solution.

This suggests a rather strong variation of the pressure with the salt concentration in the solution. Indeed, performing a large number of experiments, a strong correlation is found between the measured pressure and the salt concentration in the solution around the crystal (Fig. 5a). We use the concentration in the solution at the onset of crystal precipitation because it is the one we can determine with the greatest accuracy; Moreover, the crystal reaches the gap in the first 5 seconds and itgrows only logarithmically slowly^33^, i.e. slower than the evaporation. Also due to the slowness of the diffusion of ions (D_Na+_ ~ 1.3 10^−9^ m^2^/s in water) over the typical time scale in the experiment (~1000s) there will be little exchange between the entrapped film and the remaining solution besides the crystal; the typical size of the reservoir is ~1 cm, whereas the diffusion length is at most a fraction of a mm.

As an independent check that the salt concentration in the liquid film entrapped between the crystal and the wall is indeed high, and hence comparable to that at the onset of salt precipitation, we stop the experiment when the maximum force and maximum shear modulus are achieved but a thin liquid film is still visible under the microscope (t ~ 150 min); the concentration at the onset of precipitation was 1.4 in this experiment. The crystal is gently removed and after further drying the upper crystal face (i.e. confined face) is observed using scanning-electron microscopy (SEM). The microphotographs reveal the formation of large amount of tiny dendritic crystals (Fig. 4); these are characteristic of high supersaturation. Indeed such a kinetics of growth have been reported for supersatruation above 1.4^28^.

Assuming, then, that the concentration in the film equals the supersaturation at the onset of crystallization, we can compare with the simplified model for an electrostatic repulsion. We find that the measured pressure as a function of the supersaturation closely follows the expression for the disjoining pressure Π(l) ~ A exp(−κl), with a constant film thickness *l* of about 1.5 nm (Fig. 5a). This value is again in very good agreement with the expected magnitude for such a film (*l* ~ 2 nm)^21^,^38^. This is perhaps surprising that this simple DLVO-type expression^38^, gives such a good description of our data; both non linear terms in the Poisson-Boltzman treatment of this problem could become important at these high saturations as well as non-DLVO forces such as hydration forces at distance lower than 1 nm^26^,^39^,^40^,^41^,^42^,^43^,^44^. This merits further discussion, but is beyond the scope of this paper.

The scenario of a repulsive force requires that the crystal/solution and the solution/glass interfaces carry a charge of identical sign. Although it is known that glass surfaces are charged negatively when in contact with water and a low concentration of salt^45^, it is less evident that this remains the case if they are in contact with a highly saturated salt solution^46^,^47^,^48^. Consequently, we have determined the surface charges in highly concentrated salt solutions (i.e. close to saturation) by measuring the adsorption of cationic (Methylene Blue/C16H18ClN3S, at 8.10^−6^ M) and anionic dye (Eosine Y/C_20_H_6_Br_4_Na_2_O_5_, at 1.2.10^−5^ M) onto glass and crystals surfaces in contact with a highly concentrated salt solution. Methylene blue adsorption is widely used for the determination of both surface areas and exchange capacities of clay minerals^49^. Using a UV/Vis-Spectrometer one can measure the decoloration of a dye solution due to the adsorption of the dye on the oppositely charged surfaces (in our case on the glass surface and salt crystals), and determine in this way the nature of the charge of a given surface and its charge density (see Supplementary Information). These experiments show that glass, NaCl and KCl are all negatively charged since they adsorb the cationic dye and thus allow for the development of a repulsive disjoining pressure (Fig. 5).

More quantitatively, these measurments allows to estimate the surface charge density *σ* at the silica surface in contact with water and both NaCl and KCl solutions (see Supplementary Information). In pure water we find σ ~ 0.22 C.m^−2^ in good agreement with reported values^38^. The surface charge density of glass decreases in contact with salt solutions: σ ~ 0.12 C.m^−2^ for NaCl and σ ~ 0.072 C.m^−2^ for KCl. The lower charge density for the KCl solution is probably due to the larger size of K^+^ (ionic radius of 1.33 Å) compared to Na+ (0.96 Å), which results in smaller hydrated radii. Due to a smaller radius, the K^+^ ions bind more easily to the negatively charged silica surface at high concentrations^46^. Since the surface charge features directly in the amplitude A of the disjoining pressure, one would anticipate a much smaller crystallization pressure for KCl compared to NaCl. Indeed, if we repeat the experiments of force measurements with KCl solution, we find much smaller repulsive force (F ~ 0.008 N); in a typical experiment at the end of drying the induce repulsive pressure is P ~ 30 ± 15 MPa - roughly an order of magnitude smaller than for NaCl, in line with the significantly smaller surface charge of KCl (Fig. 3). Reflecting these lower pressures we also find much less damage when we expose pieces of sandstone to multiple dissolution/drying cycles using a KCl solution compared to NaCl^3^. Unfortunately, the range of supersaturations achieved in the several experiments with KCl was very narrow (S = 1.22 ± 0.05), which does not allow us to plot the measured pressure as a function of different concentration for this case.

Thus, the dependence of the force on salt concentration, surface charge and wettability of the glass makes a strong point that the observed forces are due to the disjoining pressure of the thin liquid film between two charged surfaces. It is interesting to compare this disjoining pressure with the thermodynamic crystallization pressure^17^,^20^. The disjoining pressure opposes the contact between the crystal and the wall by sustaining the liquid film. It is only when the magnitude of the crystallization pressure reaches the disjoining pressure that the crystal is forced into the contact with the wall by disrupting this thin film. As discussed theoretically by Scherer^21^, the disjoining pressure sets therefore an upper bound to the crystallization pressure. Our experimental results show indeed that for NaCl, for a supersaturation of S ~1.5, the calculated crystallization pressure^20^ is on the order of 135 MPa, similar to the disjoining pressure measured here (150 MPa ± 50) at this supersaturation. This means that up to this supersaturation, because of higher disjoining pressure compare to the crystallization pressure, the liquid film is sustained. Therefore the crystal can grow and exerce a crystallization pressure exceeding the tensile strength of stones (sandstone ~0.9 MPa^50^); crystallization can thus induce damage in the stone. It should be noted that salt damage becomes more visible for sodium chloride after several cycles of changing the humidity, because with cycling the recrystallization induces the precipitation of larger crystals within the pores^3^ (i.e in confinement) rather than inducing efflorescence of small microcrystallites onto the surface of the porous material. Thus the weathering due to in-pore crystallization becomes more pronounced with increasing number of cycles.

In conclusion, we have performed a direct measurement of the pressure exerted by the spontaneous precipitation of a salt microcrystal during evaporation in a confinement similar to that found in porous materials The results obtained for the growth of KCl and NaCl crystals between hydrophilic and hydrophobic glass walls suggests that the pressure originates from a repulsive interaction between charged surfaces separated by the liquid film. The magnitude of the measured pressure can be very high. The subtle dependence of the generated stress on the wetting and the surface charge properties that we report can also explain the experimentally observed variability for different salts crystallizing in different materials^16^,^23^,^24^,^25^,^26^. Hydrophobic treatments such as water repellent or consolidant products have for instance been used in the past^19^,^51^,^52^,^53^; We show here experimentally how a hydrophobic treatment that supresses the surface charge and wetting film could in principle prevent the development of crystallization pressure when salt crystallizes in the pore space (i.e. confinement). Although such treatments might help to limit the adverse effects of salt crystallization in practice, they also might could have unintended consequences with regards to other weathering mechanisms. It is worthwhile to mention that such unintended effects are likely not due to the chemical treatment itself but rather to its heterogeneity. The efficiency of hydrophobic treatments strongly depends on the impregnation depth of the chemicals in the stone, which in turn affects the wetting properties of the porous materials and modify the flow of the liquid within the porous matrix^28^,^53^,^54^,^55^,^56^. A heterogeneous hydrophobic treatment can lead to the formation of entrapped liquid pockets in untreated hydrophilic regions in the porous materials, and if salt crystallizes here this may lead to weathering. The understanding of the processes that are at the origin of alterations of porous artworks is of great importance for managing and preserving them for future generations.

## Additional Information

**How to cite this article**: Desarnaud, J. *et al*. The Pressure induced by salt crystallization in confinement. *Sci. Rep.* **6**, 30856; doi: 10.1038/srep30856 (2016).

## Supplementary Material

Supplementary Information

## References

[b1] Augustin C. Climate Change and World Heritage: Report on Predicting and Managing the Impacts of Climate Change on World Heritage and Strategy to Assist States Parties to Implement Appropriate Management Responses (Ed. UNESCO World Heritage Centre, Paris 2007).

[b2] Sabionni C., Cassar M., Brimblecome P. & Lefevre R. A. Vulnerability of cultural heritage to climate change, EUR-OPA major hazards agreement, Council of Europe November (2008).

[b3] Desarnaud J., Derlyun H., Molari L., de Miranda S., Cnudde V. & Shahidzadeh N. Drying of Salt contaminated porous media; Effect of primary and secondary nucleation. J. App. Phys. 118, 114901 (2015).

[b4] Wust R. A. & Schluster C. The origin of soluble salts in rocks of thebes mountains, Egypt: The damage potential to ancient Egyptian wall art. J. Archaeol. Sci. 27, 1161–1172 (2000).

[b5] Goudies A. S. & Viles H. A. Salt Weathering Hazard (Wiley, London, 1997).

[b6] Shahidzadeh-Bonn N., Desarnaud J., Bertrand F., Chateau X. & Bonn D. Damage in porous media due to salt crystallization. Phys. Rev. E. 81, 066110 (2010). 10.1103/PhysRevE.81.066110 20866481

[b7] Espinosa-Marzal R. & Scherer G. W. Advances in understanding damage by salt crystallization. Environ. Earth Sci. 69, 2657–2669 (2013). 10.1021/ar9002224 20214404

[b8] Flatt R. J., Caruso F., Sanchez A. M. A. & Scherer G. W. Chemomechanics of salt damage in stone. ncomms. 5823 (2014). 10.1038/ncomms5823 25208600

[b9] Noiriel C., Renard F., Doan M. L. & Grattier J. P. Intense fracturing and fracture sealing induced by mineral growth in porous rocks. Chemical Geology. 269, 197–209 (2010).

[b10] Tsui N., Flatt R. & Scherer G. W. Crystallization damage by sodium sulfate. J. Cult. Herit. 4, 109–115 (2003).

[b11] Rijners L. A., Huinink H. P., Pel L. & Kopinga K. Experimental Evidence of Crystallization. Pressure inside Porous Media. Phys. Rev. Lett. 94, 75503 (2005). 10.1103/PhysRevLett.94.075503 15783826

[b12] Verran-Tissoires M. & Prat M. Evaporation of sodium chloride solution from a saturated porous medium with efflorescence formation. J. Fluid Mech. 749, 701–749 (2014).

[b13] Lavalle J. Recherche sur la formation lente des cristaux. Compt. Rend. Acad. Sci (Paris) 36, 493–495 (1853).

[b14] Becker G. F. & Day A. L. The linear force of growing crystals, *Proc*. Washington Academy of Sciences 7, 283–288 (1905).

[b15] Taber S. The growth of crystal under external pressure. Am. J. Sci. 4–41, 532–556 (1916).

[b16] Correns C. W. Growth and dissolution of crystals under linear pressure. Discussions of the Faraday Soc. 5, 267–271 (1949).

[b17] Flatt R. J., Steiger M. & Scherer G. W. A commented translation of the paper by C. W. Correns and W. Steinborn on crystallization pressure. Environ. Geol. 52, 187–203 (2007).

[b18] Weyl P. K. Pressure solution and the of force crystallization: a phenomenological theory. J. Geophysical Res. 64, 2001–2025 (1959).

[b19] Scherer G. W. Crystallization in pores. Cem. Concr. Res. 29, 1347–58 (1999).

[b20] Steiger M. Crystal growth in porous materials: I The crystallization pressure of large crystals. J. Crys. Growth. 282, 470 (2005).

[b21] Scherer G. W. Stress from crystallization of salt in pores, *Proceedings of* 9^*th*^ *international congress on deterioration and conservation of stone*, Venice-Italy (2000).

[b22] Coussy O. Deformation and stress from in-pore drying-induced crystallization of salt. J. Mech. Phys. Solids. 54, 1517–1547 (2006).

[b23] Désarnaud J., Grauby O., Bromblet P., Vallet J. M. & Baronnet A. Growth and Dissolution of Crystal under Load: New Experimental Results on KCl. Cryst. Growth Des. 13, 1067–1074 (2013).

[b24] Sekine K., Okamoto A. & Hayashi K. *In situ* observation of the crystallization pressure induced by halite crystal growth in a microfluidic channel. American Mineralogist. 96, 1012–1019 (2011).

[b25] Royne A. & Dysthe D. K. Rim formation on crystal faces growing in confinement. J. of Crystal Growth. 346, 89–100 (2012).

[b26] Hamilton A., Koutos V. & Hall C. Direct measurement of salt mineral repulsion using atomic force microscopy. ChemComm. 46, 5235–5327 (2010). 10.1039/b915709c 20571677

[b27] Bosworth W. Strain-induced preferential dissolution of halite. TectonoPhysics. 78, 509–525 (1981).

[b28] Desarnaud J., Derluyn H., Carmeliet J., Bonn D. & Shahidzadeh N. Metastability limit for the nucleation of NaCl crystals in confinement. J. Phys. Chem. Lett. 5, 890–895 (2014). 26274084 10.1021/jz500090x

[b29] Naillon A., Duru P., Marcoux M. & Prat M. Evaporation with sodium chloride crystallization in capillary tube. J. Cryst. Growth 422, 52–61 (2015).

[b30] Shahidzadeh N. & Desarnaud J. Damage in porous media: role of the kinetics of salt (re) crystallization. EPJAP 60, 24205 (2012).

[b31] De Gennes P. G., Brochard F. & Quere D. Capillarity and Wetting Phenomena, Drops, Bubbles, Pearls, Waves (Springer Science, New York, 2004).

[b32] Shahidzadeh-Bonn N., Rafai S., Bonn D. & Wegdam G. Salt Crystallization during Evaporation: Impact of Interfacial Properties. Langmuir 24, 8599–8605 (2008). 18652495 10.1021/la8005629

[b33] Shahidzadeh N. & Desarnaud J. Salt Crystal Purification by Deliquescence/Crystallization Cycling. Euro. Phys. Lett. 95, 48002 1–6 (2011).

[b34] Hellström L. H. O., Samaha M. A., Wang K. M., Smits A. J. & Hultmark M. Errors in parallel-plate and cone-plate rheometer measurements due to sample underfill. Meas. Sci. Technol. 26, 1 (2015).

[b35] Meade C. & Jeanloz R. Yield strength of the B1 and B2 phases of NaCl. J. Geophys. Res. 93, 3270–3274 (1988).

[b36] Winkler E. M. & Singer P. C. Crystallization pressure of salt in stone and concrete. Geol Soc Am Bull 83, 3509–3514 (1972).

[b37] Winkler E. M. & Wilhelm E. J. Salt burst by hydration pressures in architectural stone in urban atmosphere. Geol Soc Am Bull 81, 567–572 (1970).

[b38] Israelchvili J. Intermolecular and surface forces [second edition] (Academic Press 1992).

[b39] Espinoza-Marzal R. M., Drobeck T., Balmer T. & Heuberger M. P. Hydrated ions ordering in electrical double layers. Phys. Chem. Chem. Phys. 14, 6085–6093 (2012). 22441032 10.1039/c2cp40255f

[b40] Veeramasuneni S. Y., Hu Y., Yalamanchili M. R. & Miller J. D. Interaction Forces at High Ionic Strengths: The Role of Polar Interfacial Interactions. J. Colloid Interface Sci. 188, 473–480 (1997).

[b41] Veeramasuneni S. Y., Yalamanchili M. R. & Miller J. D. Interactions between dissimilar surfaces in high ionic strength solutions as determined by atomic force microscopy. Colloids and Surfaces A: Physicochemical and Engineering Aspects 131, 77–87 (1998).

[b42] Veeramasuneni S., Hu Y. & Miller J. D. The surface charge of alkali halides: consideration of the partial hydration of surface lattice ions. Surface Science 382, 127–136 (1997).

[b43] Alcantar N., Israelchvili J. & Boles J. Force and ionic transport between mica surfaces: Implications for pressure solution. Geochimica et Cosmochimica Acta 67, 1289–1304 (2002).

[b44] Perkin S., Goldberg R., Chai L., Kampf N. & Klein J. Dynamic properties of confined hydration layers. Farady discuss 141, 399–413 (2009). 10.1039/b805244a 19227367

[b45] Siretanu I. . Direct observation of ionic structure at solid-liquid interfaces: a deep look into the Stern Layer. Scientific Reports 4, 4956 (2014). 24850566 10.1038/srep04956 PMC4030399

[b46] Dihson M., Zohar O. & Sivan U. From repulsion to attraction and back to repulsion: the effect of NaCl, KCl, and CsCl on the force between silica surfaces in aqueous solution. Langmuir. 25(5), 2831–2836 (2009). 19437699 10.1021/la803022b

[b47] Du H. & Miller J. D. Interfacial Water Structure and Surface Charge of Selected Alkali Chloride Salt Crystals in Saturated Solutions: A Molecular Dynamics Modeling Study. J. Phys. Chem. C. 111, 10013–10022 (2007).

[b48] Miller J. D. & Yalamanchili M. R. Surface Charge of Alkali Halide Particles As Determined by Laser-Doppler Electrophoresis. Langmuir 8, 1464–1469 (1992).

[b49] Hang P. T. & Brindley G. W. Methylene blue adsorption by clay minerals. Clays and Clay Minerals. 18, 203–212 (1970).

[b50] Pavlik Z. . Water and salt transport and storage properties of Mšené sandstone. Construction and Building Materials. 22, 1736–1748 (2008).

[b51] Wheeler G. Alkoxysilane and the consolidation of stones (Getty publications, Los Angeles 2005).

[b52] Brinker C. J. & Scherer G. W. The Physics and Chemistry of sol-Gel processing (Academic press Inc, 1990).

[b53] Brus J. & Kotlik P. Consolidation of stone by mixtures of alkoxysilane and acrylic polymer. Stud. Cons. 41, 109–119 (1996).

[b54] Mosquera M. J. & Pozo J. Stress During Drying of Two Stone Consolidants Applied in Monumental Conservation. Journal of Sol-Gel Science and Technology 26.

[b55] Illescas Juan F. & Mosquera María J. Surfactant-Synthesized PDMS/Silica Nanomaterials Improve, Robustness and Stain Resistance of Carbonate Stone. J. Phys. Chem. C 115, 14624–14634 (2011).

[b56] Shahidzadeh N. Effect of hydrophobization on wetting, drying, and salt crystallization in porous materials. Restoration of buildings and monuments 20, 1–10 (2014).

