# Supplementary material for: The Pressure induced by salt crystallization in confinement
Source: Sci Rep. 2016 Aug 5;6:30856. doi: 10.1038/srep30856 (PMC4974634; doi:10.1038/srep30856)
Supplement: Supplementary Information [file srep30856-s1.pdf]

Supporting information

The Pressure induced by salt crystallization in confinement

*J. Desarnaud, D. Bonn, N. Shahidzadeh**

Van der Waals-Zeeman Institute, Institute of Physics, University of Amsterdam, Science Park 904, 1098 XH Amsterdam, The Netherlands

Corresponding Author

*Dr. Noushine Shahidzadeh: n.shahidzadeh@uva.nl

1) Experimental procedure
2) Determination of the attractive capillary force
3) Determination of the crystal-walls contact area
4) Determination of surface charges for glass, NaCl and KCl crystals in concentrated salt solution.

1- Experimental procedure

We perform experiments with sodium chloride (NaCl) and Potassium chloride (KCl), both from Sigma Aldrich, at 99.99% purity. We prepare each salt in a solution slightly below the saturation S = c_i/c_s = 0.9 with c_i concentration of the solution and c_s concentration of the solution at saturation (i.e., c_i = 5.5 mol.kg^{-1} and c_i = 4.1 mol.kg^{-1} for NaCl and KCl, respectively). The equilibrium relative humidity of the saturated salt solutions are RH_{eq} = 75,5% for NaCl and RH_{eq} = 85,9% for KCl at T=20°.

We use a home-built innovative set-up to directly measure the force induced by a growing crystal. An inverted phase contrast microscope (Zeiss) is coupled to a rheometer (Rheoplus 301-D) inside a homemade controlled climatic chamber[1] with RH =40 ± 3% and RH=4% and the temperature T=21 ± 1ºC (Fig. 2). The tip of the geometry of the rheometer has a diameter of 12.5 mm and it is covered by a glass slide, which is either hydrophilic or hydrophobic depending on the experiment. The rheometer is used in oscillation mode with an amplitude of 0.01 and a frequency of 0.5 Hertz. It records a value of normal force and apparent elastic modulus during the whole experiment time t=300 min.

A very small volume of the salt solution (NaCl or KCl) is deposited on a cleaned glass slide on the inverted microscope. The second glass slide attached to the rheometer tip is used to confine the drop from above with a microscale gap (50 μm). By direct visualization using a CCD camera coupled to the inverted optical microscope, we have followed during the evaporation, the volume change of the entrapped salt solution, the onset of the crystal precipitation, and the crystal growth

in the solution. Processing of the data allows to determine the evaporation rate, the concentration of the solution at the onset of crystallization and the growth rate of the crystal.

At the end of drying the microcrystal is gently removed and analysed with Scanning Electron Microscopy (TM Hitachi 3000).

The experiments are performed in between both hydrophilic (untreated) and hydrophobic (silanised) standard ®Metzel glass slides. The hydrophilic slides consist of standard glass slides cleaned with ethanol and Millipore water and dried under nitrogen. The hydrophobic slides were prepared using Octyltriethoxysilane (Dynasylan® OCTEO) for silanisation: the cleaned glass slides are immersed in a solution composed of (1%wt OCTEO, 2%wt pure water, 0.2%wt HCl and ~97%wt isopropanol) for 30 min and subsequently dried at ~70° for one hour. The surface tension of the prepared salt solutions and their contact angles with hydrophilic and hydrophobic glass plates are measured using a Kruss instrument.We find: γ_{lv}~84 mN/m and γ_{lv}~80mN/m for the NaCl (5.5 mol.kg^{-1}) and KCl (4.10 mol.kg^{-1}) respectively. The measure contact angles are the following: θ_{NaCl} ~50º and θ_{KCl} ~45º on hydrophilic glass plates and θ_{NaCl} ~110º and θ_{KCl} ~106º on hydrophobic ones.

2- Determination of the attractive capillary force

The attractive capillary force (F_C) between the two glass plates due to the entrapped volume of salt solution is calculated from Equation (1)[2] and the images recorded during evaporation.

$$Fc = \pi R^2 \frac{2\gamma cos\theta}{H} \qquad (1)$$

where R is the radius of the droplet, γ the liquid/vapor surface tension of the salt solution, H the gap between the 2 glass slides (50 μm) and θ the contact angle of the salt solution with the glass slide. The calculated capillary force is about 4 ± 10^{-3} N, and decreases with time in good agreement with experimental values obtained using the rheometer ~-3.5 10^{-3} N.

3- Determination of the crystal-walls contact area.

We have imposed very small oscillations (an amplitude of 0.01 and a frequency of 0.5 Hertz) to the upper plate connected to the rheometer in order to measure the resistance to a shear force of the material between the two plates and consequently deduce the surface area of the salt crystal that is contact with the plates; the salt solution is a liquid that does not resist shear, whereas the salt crystal does. The rheometer gives the shear modulus of any solid material that occupies the gap between the two plates, by measuring the shear stress necessary for a certain imposed deformation. For the case of a single material in between glass plates, the shear stress depends on its elastic modules as follows:

$$\sigma = G\gamma \quad , \qquad (2)$$

with σ the stress, γ the strain, and G the elastic modulus (G_{NaCl} ~ 12.61 10^{9} Pa and G_{KCl} ~ 6.24 10^{9} Pa[5]). This expression assumes that the entire space between the two plates is filled with the solid. Consequently, the ratio of the apparent (G_{app}) to the real shear modulus is then directly the surface area of the salt crystal that is in contact with the plates:

$$\sigma = \frac{F}{A} = \frac{F}{\pi . R_{plate}^{2}} = G_{app}\gamma \qquad (3)$$

$$\sigma = \frac{F}{A_{crystal}} = G_{NaCl}\gamma \qquad (4)$$

with F the normal force, A the surface area in contact with the glass slides, $A_{crystal}$ the surface area of the crystal in contact with the glass slides, R the radius of the upper plate (12.5 10^{-2} m), G_{app} the apparent elastic modulus, G_{real} the shear modulus of NaCl crystal.

$$\frac{G_{app}}{G_{NaCl}} = \frac{F}{\pi.R_{plate}^2} \cdot \frac{Acrystal}{F} \qquad (5)$$

$$G_{app} = \frac{A_{crystal}}{\pi.R_{plate}^2} \cdot G_{real} \qquad (6)$$

From these equations we can conclude that:

$$A_{crystal} = \frac{G_{app}.\pi.R_{plate}^2}{G_{crystal}} \qquad (7)$$

4- Determination of glass, NaCl, and KCl crystals surface charges in concentrated salt solution.

Using UV-Vis spectrometry, we experimentally determine the sign of surface charges on glass, NaCl crystals and KCl crystals in contact with a high salt concentration solution (i.e., at saturation NaCl = 6.16 mol.kg^{-1}, KCl = 4.56 mol.kg^{-1}). We use two dyes: a cationic dye (methylene blue / C16H18ClN3S, at 8.10^{-6} M), which has a maximum absorbance band at 660 nm, and an anionic dye (Eosine Y / $C_{20}H_6Br_4Na_2O_5$, at 1.2.10^{-5} M), which has a maximum absorbance band at 530 nm[4,5].

In the case of cationic dye, the experiments show that the addition of glass beads and NaCl crystals to the reference solution reduce the absorbance by 30% and 40% respectively (Figure 4b). Conversely no variation in absorbance is measured with the addition of silanised glass beads. In the experiments done with the anionic die, no change in the absorbance is detected after the addition of glass beads or silanised glass beads or NaCl crystals.

These results indicate that both glass and crystal surfaces are negatively charged whereas silanised glass beads are not.

Consequenlty, we have calculated the charge density thank to the Beer-Lambert law.

$$A_{\Lambda} = \varepsilon_{\Lambda}.l.C$$

With A_{Λ} the absorbance of the material measured by the spectrometer, ε_{Λ} the molar attenuation coefficient, l the path length of the beam light through the material, C the amount concentration of the attenuating species in the material. The attenuation coefficient has been measured for a serie of Methylene blue solution with different concentration of NaCl (from 0 to 5.8 $mol.kg^{-1}$) and KCl (from 0 to 4.5 $mol.kg^{-1}$). We have made a calibration curve by measuring first the absorbance of methylene blue in different solutions with known salt concentrations and then the absorbance after adding a given amount of glass beads in these solutions (2g). in Figure S1 the difference between these two absorbance for each salt concentration is plotted. The latter shows that for concentrations higher than 1Molal, the reduction of the absorbance band due to the addition of glass beads remains constant (Figure S1). Consequently, the concentration of the MB in the solution and the adsorbed amount MB on glass beads is calculated .

Because we knew the specific surface area of the beads added to the solution we have calculated the number of mole of methylene blue adsorbs by square meter and then the number of methylene blue molecule adsorbs at the surface. The elementary charge, the charge of a proton (equivalently, the negative of the charge of an electron) being approximatively 1.6 x 10^{-19} C we have calculated the charge density of the glass beads in Cm^2 in contact with a NaCl and also KCl solution with different concentration.

We find σ ~ 0.22 $C.m^{-2}$ in water, σ ~ 0.12 $C.m^{-2}$ in NaCl solution and σ ~ 0.072 $C.m^{-2}$ for KCl solution.

In the case of KCl and the cationic dye, the reduction of the absorbance band is less after the addition of glass beads (8%) or KCl crystals (26%) to the reference solution. No variation in absorbance is measured with the addition of silanised glass beads. The experiments done with the anionic dye reveal no variation in absorbance with the addition of glass beads, silanised glass beads or KCl crystals. Therefore, we conclude that KCl crystals in contact with KCl-saturated solution (4.56M) are negatively charged, although the charge density of the glass beads in contact with a KCl-saturated solution seems to be much lower than in contact to a NaCl saturated solution. As it was shown previously, larger cations (K^+ has an ionic radius of 1.33 Å which is larger than that of Na^+, 0.96Å) can bind more easily to silica surfaces at high concentrations[6].

5- Supporting pictures

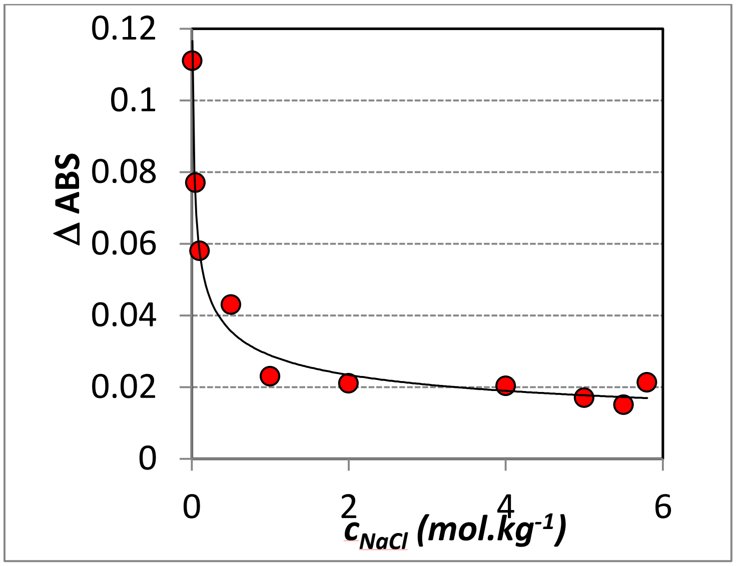

Figure S1: The difference in the absorbance of Methylene blue ΔABS for different solute concentrations C_{NaCl} before and after the addition of glass beads.

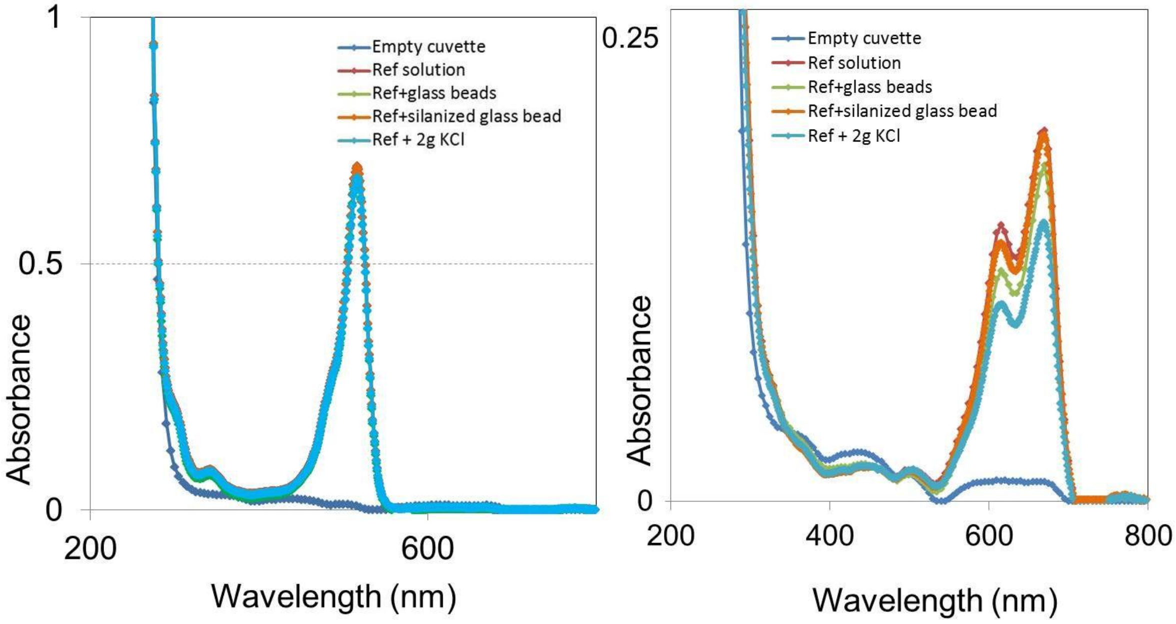

Figure S2: Spectrometer UV-Vis experiments for KCl-EosineY solution (Left) and KCl methylene blue solution (Right) in contact with cleaned, silanised glass beads and KCl crystals. Ref solution corresponds to KCl- EosineY (left) or KCl-methylene blue (right).

References

1. Shahidzadeh, N. & Desarnaud, J. Damage in porous media: Role of the kinetics of salt (re)crystallization. *Eur.Phys. J. Appl. Phys. 60, 242025-1-7* (2012)
2. De Gennes, P.G, Brochard, F. & Quere, D. *Capillarity and Wetting Phenomena, Drops, Bubbles, Pearls, Waves* (Springer Science, New York, 2004).
3. Handbook of Chemistry and Physics. 85th edition CRC PRESS, NYC, 2005
4. Bahney, C.S, Lujan, T.J., Hsu, C.W. M., Bottlang, West , J.L., and Johnstone. & B. Visible light photo initiation of mesenchymal STEM cell-laden bioresponsive hydrogels. *Eur. Cell. Mat.* 22, 43-55 (2015)
5. Chen. G., Pan, J., Han, B. & Yan. H. Absoption of methylene blue on Montmorillonite. J. Dispersion. Science and Technology. 20, 1179-1187 (1999)
6. Dihson, M., Zohar, O. & Sivan, U. From repulsion to attraction and back to repulsion: the effect of NaCl, KCl, and CsCl on the force between silica surfaces in aqueous solution, *Langmuir.* 25, 2831-6 (2009)
